# Targeting GLP-1 receptors for repeated magnetic resonance imaging differentiates graded losses of pancreatic beta cells in mice

**DOI:** 10.1007/s00125-014-3442-2

**Published:** 2014-11-22

**Authors:** Laurent Vinet, Smaragda Lamprianou, Andrej Babič, Norbert Lange, Fabrizio Thorel, Pedro Luis Herrera, Xavier Montet, Paolo Meda

**Affiliations:** 1 Department of Genetic Medicine and Development, University of Geneva, Geneva, CMU, 1 rue Michel-Servet, CH-1211 Geneva 4, Switzerland; 2 School of Pharmaceutical Sciences, University of Geneva, University of Lausanne, Geneva, Switzerland; 3 Department of Radiology, Geneva University Hospital, Geneva, Switzerland; 4 Department of Cell Physiology and Metabolism, University of Geneva, Geneva, Switzerland

**Keywords:** Beta cell mass, Exendin, Glucagon-like peptide 1 receptors, Magnetic resonance imaging, Multimodality probe, Targeted iron oxide nanoparticles

## Abstract

**Aims/hypothesis:**

Non-invasive imaging of beta cells is a much-needed development but is one that faces significant biological and technological hurdles. A relevant imaging method should at least allow for an evaluation over time of the mass of beta cells under physiological and pathological conditions, and for an assessment of novel therapies. We, therefore, investigated the ability of a new MRI probe to repeatedly measure the loss of beta cells in a rodent model.

**Methods:**

We developed an innovative nanoparticle probe that targets the glucagon-like peptide 1 receptor, and can be used for both fluorescence imaging and MRI. Using fluorescence, we characterised the specificity and biodistribution of the probe. Using 1.5T MRI, we longitudinally imaged the changes in insulin content in male and female mice of the RIP-DTr strain, which mimic the changes expected in type 1 and type 2 diabetes, respectively.

**Results:**

We showed that this probe selectively labelled beta cells in situ, imaged in vivo native pancreatic islets and evaluated their loss after diphtheria toxin administration, in a model of graded beta cell deletion. Thus, using clinical MRI, the probe quantitatively differentiates, in the same mouse strain, between female animals featuring a 50% loss of beta cells and the males featuring an almost complete loss of beta cells.

**Conclusions/interpretation:**

The approach addresses several of the hurdles that have so far limited the non-invasive imaging of beta cells, including the potential to repeatedly monitor the very same animals using clinically available equipment, and to differentiate graded losses of beta cells.

**Electronic supplementary material:**

The online version of this article (doi:10.1007/s00125-014-3442-2) contains peer-reviewed but unedited supplementary material, which is available to authorised users.

## Introduction

The non-invasive, repeated in vivo imaging of beta cells, which are central players in most forms of diabetes [1], is highly desirable from both a research and a clinical standpoint. The method should allow for the evaluation of the normal mass and function of beta cells, of their spontaneous changes with time under physiological and pathological conditions and of the effects of candidate therapies [2]. The development of such an imaging method, which is complicated by a combination of anatomical, biological and technical hurdles [2, 3], may be eased by the use of sensitive and beta cell-specific probes. The glucagon-like peptide 1 receptor (GLP-1r), which has become an important target in the treatment of type 2 diabetes [4], is emerging as a potential target for such probes, because of its high expression in beta cells [5]. The endogenous ligand of the GLP-1r (i.e. GLP-1), induces the release of insulin in a glucose-dependent manner and promotes beta cell proliferation in adult rodents [6]. The long-acting analogue exendin-4 also binds to the extracellular domain of GLP-1rs with picomolar affinity [7], and exendin derivatives have been developed for fluorescence, nuclear and magnetic resonance imaging of beta cells [8–11], transplanted islets [12, 13] and insulinomas [14–16] and for screening of patients with type 1 diabetes [17].

The position of the pancreas within the abdomen and the dispersion of its small endocrine islets imply the need for a deep-penetrating and highly sensitive approach. Therefore, most of these studies have relied on nuclear medical imaging methods (positron emission computed tomography [PET] and single photon emission computed tomography [SPECT]), in spite of the limited spatial resolution of these methods and their potential safety issues [2, 3]. Specifically, the radiation dose might be a potential limiting factor in longitudinal studies of healthy volunteers and diabetic patients, as pointed out in several studies with radiolabelled exendin [11, 14, 18, 19]. In the search for alternatives, several studies have investigated MRI [20–22], a method that does not involve ionising radiation. The modality provides for an unsurpassed anatomical visualisation of soft tissues, including individual islets in experimental models when high magnetic fields are used [21, 23]. Still, MRI has a significantly lower sensitivity than either PET or SPECT, and hardly allows for an absolute quantitative evaluation of the signal [2, 3]. The development of targeted ultrasmall superparamagnetic particles of iron oxide (USPIO) [24–26], nanoparticles that modify the magnetic resonance signal [27–29], may help to ameliorate some of these limitations. Thus, a recent study has documented that such MRI probes permit the in vivo differentiation of normoglycaemic from hyperglycaemic animals in a rodent model of type 1 diabetes [30]. To assess whether this approach could be adapted to the repeated, sequential monitoring of individual animals, specifically under conditions of a partial loss of beta cells such as is expected in type 2 diabetic patients [31], we developed a new, dual modality nanoparticle probe that targets the GLP-1r and that is suitable for both fluorescence and MRI. Here, we show that this probe permits the following: (1) selective labelling of beta cells in situ; (2) imaging of native pancreatic islets in vivo; (3) repeated and longitudinal monitoring of individual animals and (4) differentiation of animals with a partial loss of beta cells from those with an almost complete loss.

## Methods

### Synthesis of the exendin–nanoparticle probe

**Fig. 1:**
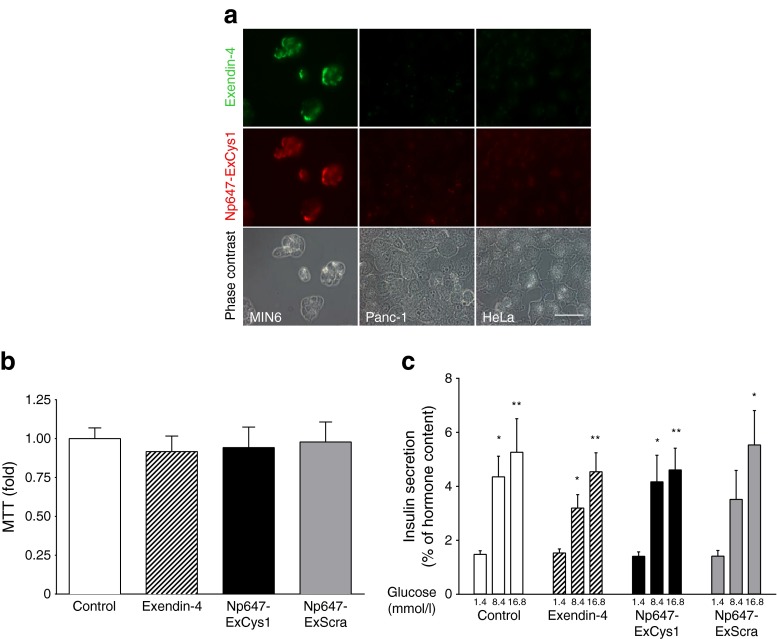
(**a**) Fluorescence microscopy of MIN6, Panc-1 and HeLa cells after 30 min incubation in the presence of Np647–ExCys1. Scale bar, 50 μm. (**b**) MTT assay on MIN6 cells exposed for 24 h to exendin-4, Np647–ExCys1 or Np647–ExScra (*n* = 3 per condition; each condition was expressed as the fold change relative to control set to 1). (**c**) Glucose-stimulated insulin secretion of MIN6 cells exposed for 24 h to exendin-4, Np647–ExCys1 or Np647–ExScra (*n* = 6 per condition). Data are mean + SEM. *p < 0.05 and **p < 0.01 vs cells exposed to 1.4 mmol/l glucose

A Cys-1 modified peptide comprising 39 amino acids of exendin-4 (ExCys-1; Cis-His-Gly-Glu-Gly-Thr-Phe-Thr-Ser-Asp-Leu-Ser-Lys-Gln-Met-Glu-Glu-Glu-Ala-Val-Arg-Leu-Phe-Ile-Glu-Trp-Leu-Lys-Asn-Gly-Gly-Pro-Ser-Ser-Gly-Ala-Pro-Pro-Pro-Ser) was synthesised by GeneCust Europe (Dudelange, Luxembourg). Molday ION C6Amine iron oxide nanoparticles, featuring ~9,000 iron atoms per particle and a 35 nm diameter (Biopal, Worcester, MA, USA), were reacted with the succinimidyl ester of Alexa Fluor 647 (LifeTechnologies Europe, Zug, Switzerland) to obtain a ratio of 20 fluorochrome molecules/nanoparticle (Np647). Np647 was reacted with sulfosuccinimidyl-4-(*N*-maleimidomethyl) cyclohexane-1-carboxylate (Sulfo-SMCC; Thermo-Scientific, Rockford, IL, USA) to obtain maleimide-activated Np647, which was reacted with ExCys1 (electronic supplementary material [ESM] Fig. 1). The resulting Np647–ExCys1 conjugates were purified by size exclusion PD10 chromatography. The peptide-to-iron ratio (~25 ExCys1/nanoparticle), was determined by bicinchoninic acid protein assay, using an exendin standard curve in the presence of Np647. To control the specificity of the Np647–ExCys1 probe, we tested the following: (1) a non-targeted probe (Np647–ExScra) produced by reacting the maleimide-activated Np647 with a Cys-1 scrambled peptide (ExScra) of exendin-4 (GeneCust Europe) and (2) Np647–ExCys1 (5 μg i.v.) co-injected with free exendin-4 (750 μg s.c.; 12 h later a second s.c. injection of free exendin-4 was given to maintain a large [~100-fold] molar excess of free exendin-4 over the equivalent Np647–ExCys1 dose).

### In vitro studies

Cells of the MIN6, Panc-1 and HeLa lines were cultured as previously reported [32]. At the time of the experiments, cultures had a density of about 10^6^ cells/ml. To assess the specificity of the cell labelling, medium was removed and cells were rinsed twice in PBS and then exposed at 37°C to 50 μg/ml Np647–ExCys1 added to a Krebs Ringer bicarbonate medium. Thirty minutes later, the cells were rinsed twice in PBS and examined under an Axiophot fluorescence microscope (Zeiss, Oberkochen, Germany) equipped with filters for Alexa 647.

To assess whether Np647–ExCys1 affected the viability of MIN6 cells, cultures were exposed for 24 h to 50 μg/ml Np647–ExCys1 added directly to DMEM (Gibco-LifeTechnologies Europe, Zug, Switzerland). Cells were then tested in a thiazolyl blue tetrazolium bromide (MTT) assay (M2128; Sigma-Aldrich, St Louis, MO, USA), and for glucose-induced insulin secretion (for details, see ESM Methods).

### Rat insulin promoter-diphtheria toxin receptor mice

Mice were housed in a conventional animal facility and all animal experiments were conducted as per the protocols authorised by our institutional and State authorities (Authorisation no. 1034/3550/2).

Rat insulin promoter-diphtheria toxin receptor (RIP-DTr) mice were generated as reported [33]. Briefly, the transgene containing an insulin promoter and the diphtheria toxin (DT) receptor coding sequence (RIP-DTr) was targeted to the *Hprt* locus of the X chromosome. Thus, in this model, DT administration leads to a parallel loss of insulin content and beta cells, which is partially due to the random X inactivation (on average 50%) in hemizygous female mice and nearly complete ablation in male mice.

To evaluate the total insulin content of the pancreas, whole glands were carefully dissected and extracted in acid-ethanol for 24 h, as reported [34, 35]. Pancreas insulin content was evaluated using a rodent insulin ELISA kit (Mercodia, Uppsala, Sweden) according to the manufacturer’s instructions.

### Biodistribution of the exendin–nanoparticle probe

Male RIP-DTr mice [33] were injected through the retro-orbital venous plexus with 5 μg/(g body weight) of either Np647–ExCys1 or Np647–ExScra or were co-injected with 5 μg Np647–ExCys1 and two subcutaneous doses of 750 μg free exendin-4 at a 12 h interval (the subcutaneous route was chosen to slow down the absorption of the free peptide into the circulation).

Mice were killed 24 h later, and immediately perfused via the left ventricle, first with 10 ml 0.9% NaCl (154 mmol/l) and then with 10 ml 4% paraformaldehyde in 0.1 mol/l phosphate buffer at 37°C. The pancreas, liver, spleen, kidneys, lung, duodenum and heart were harvested and fixed in paraformaldehyde for 2 h at 4°C. The organs were rinsed for 2 h in phosphate buffer at 4°C, and their fluorescence recorded with an IVIS Spectrum (PerkinElmer, Waltham, MA, USA) equipped with filters for Alexa 647. Corresponding organs from mice injected with Np647–ExCys1 and Np647–ExScra, as well as from mice injected with Np647–ExCys1 with and without an excess of exendin-4, were imaged in parallel. To differentiate between tissue autofluorescence and fluorescence due to the A647 fluorochrome, the organs were excited using 535, 570, 605 and 640 nm excitation filters and fluorescence was recorded using a 680 nm emission filter. Spectral unmixing was performed with the Living Image 4.3.1 software (PerkinElmer, Waltham, MA, USA) and fluorescence signals (expressed as average radiant efficiency, 10^7^ [p s^–1^ cm^–2^ sr^–1^]/[μW/cm^2^]) were quantified on the unmixed image after suppression of the autofluorescence levels.

The fixed pancreas, liver, spleen and kidneys were also rinsed for 15 h in 30% sucrose, embedded in OCT compound (Sakura Finetek, Torrance, CA, USA), and cryo-sectioned at 7 μm thickness. Sections were mounted and examined by fluorescence microscopy. Pancreas sections were immunolabelled using either guinea pig antibodies against insulin (Ventrex Laboratories, Portland, ME, USA), diluted 1/200, mouse antibodies against glucagon (Sigma-Aldrich), diluted 1/2,000, or rabbit antibodies against exendin-4 (Abcam, Cambridge, UK), diluted 1/100. Secondary antibodies were anti-guinea pig antibodies coupled to Dylight 405 (Jackson Immunoresearch Laboratories, WestGrove, PA, USA) diluted 1/800, anti-mouse antibodies coupled to tetramethylrhodamine (SouthernBiotech, Birmingham, AL, USA), diluted 1/500, and anti-rabbit antibodies coupled to A488 (Molecular Probes, Eugene, OR, USA), diluted 1/500, for insulin, glucagon and exendin-4 immunolabelling, respectively. Coverslips were placed over the sections and the sections were photographed with an Axiophot fluorescence microscope (Zeiss).

### MRI

Three-to-four-months-old male and female mice of the RIP-DTr strain [33, 36] were tested before and after DT administration. Mice were initially imaged before the injection of a probe to obtain a baseline T2 value (ESM Fig. 2). The mice were then injected through the retro-orbital venous plexus with 5 mg/kg Np647–ExCys1 and were immediately imaged (time 0) for evaluation of the vascular volume fraction, which reflects the blood perfusion of the pancreas. A third MRI scan was performed 24 h later, to allow for the evaluation of the probe accumulation. One week after this first imaging session, the same mice were given three intraperitoneal injections of 125 ng DT (injections administered on days 7, 9 and 11; ESM Fig. 2) and were monitored for non-fasting blood glucose by measuring tail-vein blood samples with a glucose meter. One week after the beginning of the DT administration, the mice underwent a second imaging session, as per the protocol mentioned above (ESM Fig. 2). Female mice underwent a similar, third MRI session 3 months after the DT injection (ESM Fig. 2).

To test whether the probe accumulation reflected the insulin content of the pancreas, a group of RIP-DTr mice was killed for pancreas sampling and determination of insulin content, immediately after a single MRI session.

All magnetic resonance images were acquired on a 1.5 T clinical scanner (Philips Medical System, Best, the Netherlands), equipped with a surface coil (Ø = 47 mm). Coronal T2 maps were generated using a three-dimensional turbo spin echo (TSE) sequence with the following parameters: repetition time (TR) = 145 ms; six different echo time (TE) values ranging from 9 to 54 ms; flip angle = 90°; matrix size = 116 × 83; number of average = 1; field of view = 60 × 41; slice thickness = 0.5 mm. A total of 102 sequential coronal images were obtained to cover the entire pancreatic region. All images were analysed with the dicom viewer Osirix (Pixmeo SARL, Bernex, Switzerland). Regions of interest for analyses were manually defined on three consecutive MRI slices of each pancreas. Probe accumulation was given by log_*e*_ (T2 before nanoparticle injection/T2 24 h after nanoparticle injection) [27, 29].

### Statistical analysis

Data were analysed using either paired or unpaired Student’s *t* test, assuming a two-tailed distribution, or two-way ANOVA, followed by the Bonferroni multiple comparison post hoc test (GraphPad Prism 5; GraphPad Software, La Jolla, CA, USA). A *p* value of ≤0.05 was considered statistically significant.

## Results

### The Np647–ExCys1 probe targets insulin-containing cells, and is not toxic in vitro

Thirty minutes after the addition of 50 μg/ml Np647–ExCys1 to the culture medium of MIN6 cells, which express insulin and GLP-1r, fluorescence microscopy revealed co-staining of most cells for exendin-4 and Np647–ExCys1 (Fig. 1a). Under identical conditions, no such staining was observed in cultures of Panc-1 and HeLa cells, neither of which express insulin or GLP-1r (Fig. 1a). After 24 h exposure to 50 μg/ml of either Np647–ExCys1 or Np647–ExScra, the viability of MIN6 cells was not altered, as indicated by an MTT assay (Fig. 1b) and also by the retention of a control glucose-induced insulin secretion (Fig. 1c).

### The Np647–ExCys1 probe targets in vivo several organs expressing GLP-1rs

**Fig. 2:**
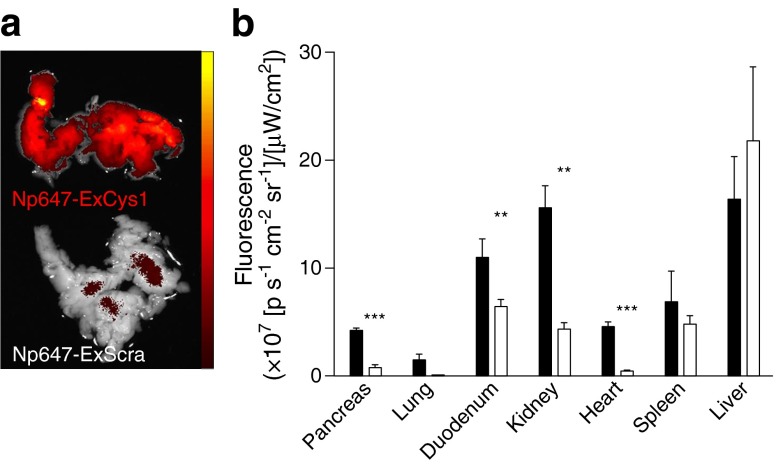
(**a**) Ex vivo fluorescence images of pancreas, 24 h after the intravenous injection of either Np647–ExCys1 or Np647–ExScra. (**b**) Semi-quantitative analysis of fluorescence 24 h after the intravenous injection of either Np647–ExCys1 (black bars) or Np647–ExScra (white bars). Data are mean + SEM (*n* = 3 mice per condition). **p < 0.01 and ***p < 0.001 vs Np647–ExCys1

Twenty-four hours after intravenous injection, the Np647–ExCys1 probe distributed in the pancreas (Fig. 2 a, b), and other organs expressing GLP-1rs, including lung, duodenum, kidney and heart (Fig. 2b). In these organs the accumulation of the non-targeted Np647–ExScra probe was lower. In contrast, the accumulation of the Np647–ExCys1 and the Np647–ExScra probes was comparable in liver and spleen, which non-specifically take up nanoparticles (Fig. 2b). An excess of free exendin-4 also significantly reduced the accumulation of Np647–ExCys1 in the organs expressing GLP-1rs, but not in liver and spleen (ESM Fig. 3a).

### The Np647–ExCys1 probe targets insulin-containing cells in vivo

**Fig. 3:**
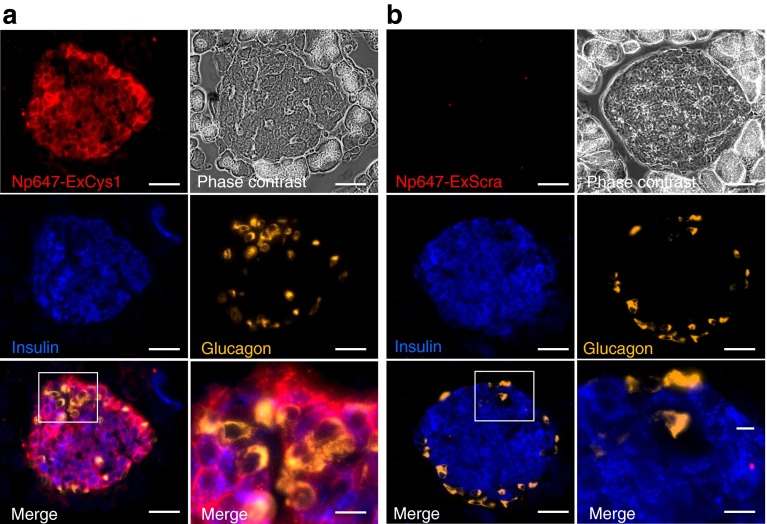
Fluorescence microscopy of control RIP-DTr pancreas sections 24 h after the intravenous injection of either Np647–ExCys1 (**a**) or Np647–ExScra (**b**). Fluorescent nanoparticles are seen in red and the pancreas sections were stained for insulin (blue) and glucagon (yellow/orange). The white box outlines the area that is shown at a higher magnification in the right merge panel. Scale bar, 30 μm in all panels, except for 10 μm in the enlargements of the boxed areas

Twenty-four hours after the intravenous injection of Np647–ExCys1 in normoglycaemic mice of the RIP-DTr strain, fluorescence microscopy revealed that the nanoparticles labelled most insulin-containing beta cells, but not the glucagon-containing alpha cells, and the surrounding acinar cells (Fig. 3a). The Np647–ExCys1 probe distributed in a similar manner to the exendin-4 peptide (ESM Fig. 4a). The beta cell labelling by nanoparticles was not observed when mice were injected with the Np647–ExScra probe (Fig. 3b) or with the Np647–ExCys1 probe in the presence of an excess exendin-4 (ESM Fig. 3d).

### The Np647–ExCys1 probe decreases the enhancement of T2-weighted magnetic resonance images

**Fig. 4:**
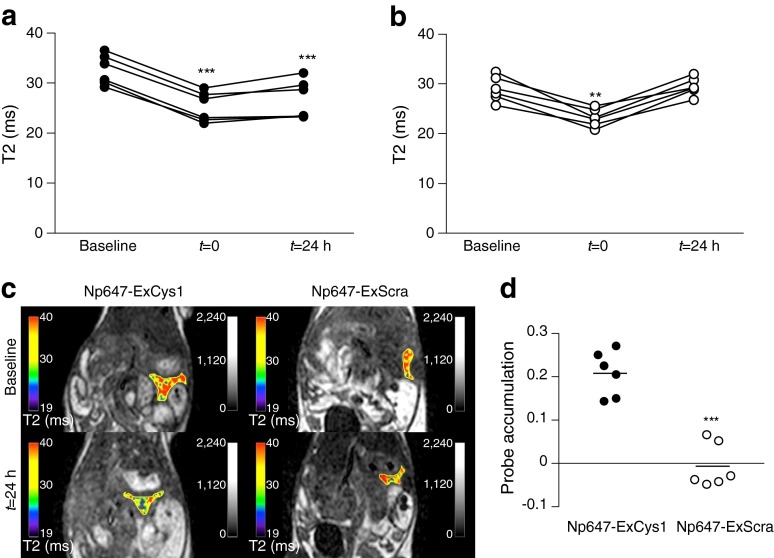
(**a**, **b**) T2 measurements before (baseline), immediately after (*t* = 0) and 24 h after the intravenous injection of either Np647–ExCys1 (**a**) or Np647–ExScra (**b**). Symbols show successive measurements in the same mice vs baseline value, **p < 0.01 and ***p < 0.001. (**c**) Representative greyscale T2-weighted magnetic resonance images of male RIP-DTr mice. A colour-coded T2 map (scale in ms on the left) is superposed on the greyscale magnetic resonance images. (**d**) Pancreatic probe accumulation in control RIP-DTr mice 24 h after the intravenous injection of either Np647–ExCys1 or Np647–ExScra. The probe accumulation is given by [log_*e*_ (T2_baseline_/T2_24 h_)]. Mean values are shown by the lines. ***p < 0.001 vs Np647–ExCys1

Immediately after the intravenous injection of either Np647–ExCys1 or Np647–ExScra (*t* = 0), the T2 of pancreas was significantly reduced compared with the baseline value measured before the injection (Fig. 4 a, b), consistent with a similar vascular distribution of the two probes. For Np647–ExScra, this change was reverted 24 h later, because of the washout of the control probe (Fig. 4b). In contrast, it was maintained for Np647–ExCys1 (Fig. 4a), consistent with the binding of the probe to the GLP-1r of beta cells. Comparison of the T2 values measured before and 24 h after the probe injection (Fig. 4c), revealed a larger pancreatic accumulation of the Np647–ExCys1 than of the Np647–ExScra probe (Fig. 4d). The accumulation of the Np647–ExCys1 probe was also decreased in the presence of an excess of free exendin-4 (ESM Fig. 3b).

### The Np647–ExCys1 probe differentiates graded losses of insulin in RIP-DTr mice

**Fig. 5:**
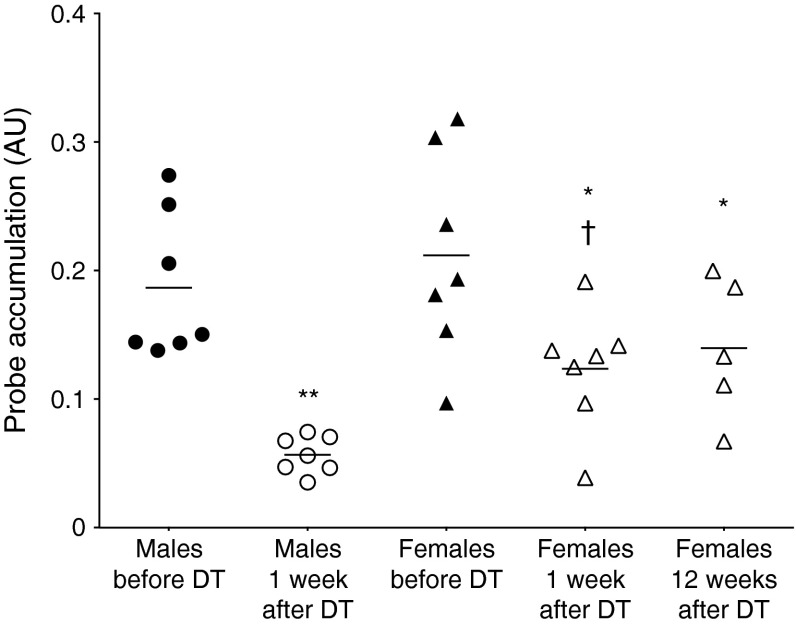
Pancreatic probe accumulation in male and female RIP-DTr mice before and after DT treatment to ablate beta cells. Symbols represent individual mice. Mean values are shown by the lines. *p < 0.05 and **p < 0.01 vs before DT for the same sex; ^†^ *p* < 0.05 vs DT-treated male mice

**Fig. 6:**
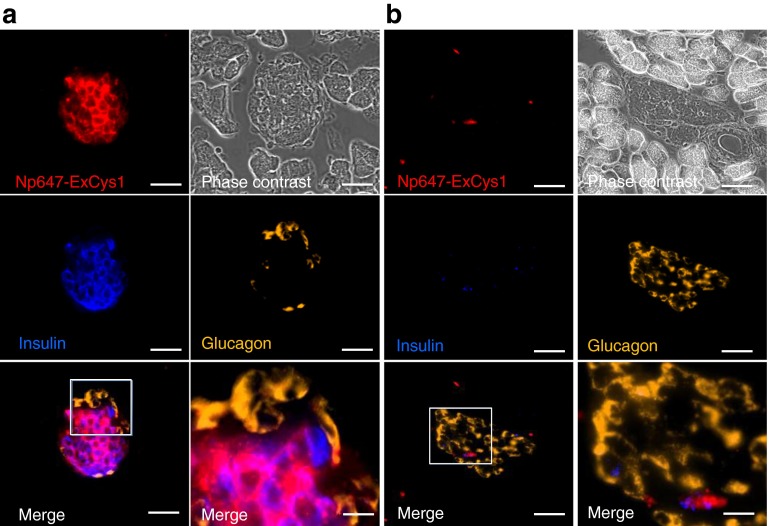
Fluorescence microscopy of pancreas sections 24 h after an intravenous injection of Np647–ExCys1 in DT-treated RIP-DTr female (**a**) and DT-treated male RIP-DTr (**b**) mice. Fluorescent nanoparticles are seen in red, and the pancreas sections were stained for insulin (blue) and glucagon (orange). The white box outlines the area that is shown at a higher magnification in the right merge panel. Scale bar, 30 μm in all panels, except for 10 μm in the enlargements of the boxed areas

We used the Np647–ExCys1 probe to repeatedly monitor the same RIP-DTr mice before and after DT administration (ESM Fig. 2). Prior to this administration, male and female RIP-DTr mice showed a similar level of pancreatic accumulation of the probe (Fig. 5). One week after DT administration, the same imaging protocol revealed a significant drop in the probe accumulation in all male mice (*p* < 0.01) and most female mice (*p* < 0.05; ESM Figs 5 and 6). This drop was significantly higher (*p* < 0.05) in male mice (average 74%) than in female mice (average 42%; Fig. 5), which associated with a sex difference in beta cells (Fig. 6) and insulin content (ESM Fig. 5a). Thus, whereas female RIP-DTr mice featured a sizeable number of beta cells (Fig. 6a) and a relatively high insulin content (on average 63% of the control levels; ESM Fig. 5a) up to 3 months after the DT administration, male mice featured a rapid, and close to complete, loss of both beta cells (Fig. 6b) and insulin (0.4% of control levels; ESM Fig. 5a). Plotting the data from a group of mice in which the pancreases were sampled immediately after the MRI session revealed a significant (*p* < 0.01) correlation (*R* = 0.834), between insulin content and the accumulation of the probe in the pancreas (ESM Fig. 5b).

## Discussion

We developed a novel probe which couples USPIOs to a modified exendin peptide in a different manner to that previously reported [30]. The new probe allows for a specific conjugation of the peptide to the iron oxide nanoparticles, while preserving the binding motif of exendin-4 for the GLP-1r [8, 37, 38]. Here, we show that the new probe, which does not alter the viability or secretory function of insulin-producing cells in vitro, selectively targets the vast majority of the endogenous beta cells in situ, even though it also binds to other abdominal organs expressing GLP-1rs. The specificity of the probe for these receptors was demonstrated ex vivo by a decreased fluorescence signal in the organs expressing GLP-1rs [39, 40], using either nanoparticles tagged with an exendin-4 scrambled peptide or exendin-4-targeted nanoparticles in the presence of an excess of free exendin-4. An exception was the heart, in which the signal was not decreased in the presence of an excess of exendin-4, possibly because the immediate and large blood perfusion of the organ resulted in a probe availability for which the free peptide could not compete. In addition*,* the beta cell specificity of the pancreatic MRI signal was demonstrated in vivo by a decreased probe accumulation in the following situations: (1) when using nanoparticles tagged with the scrambled peptide, a widely used control in nanoparticle studies [24–26, 41, 42]; (2) when using exendin-4-tagged nanoparticles in the presence of an excess of free exendin and (3) after the selective deletion of the insulin-producing cells. The pancreatic uptake of our probe was decreased by about 74% after the near-total loss of insulin in male RIP-DTr mice, which provides a larger imaging change than that recently reported in another model (about 25%) [30]. What causes the residual signal seen in these animals remains to be determined and may be attributable to the longer persistence of the nanoparticles in the blood circulation and/or their uptake by pancreatic immune cells of the diabetic mice. We further document that, despite the limited sensitivity of clinical MRI, the accumulation of the probe was sufficient to modify T2-weighted magnetic resonance images of the murine pancreas and an iron oxide dose lower than those tested in previous studies [27, 29, 30]. Most significantly, we show here that this approach allowed for the repeated monitoring and quantitative evaluation of both maximal and submaximal losses of insulin as experimentally induced in a mouse model of cell-specific deletion [33, 36]. Thus, the probe allowed differentiation of the major loss of insulin observed in male RIP-DTr mice, which parallels that of beta cells and mimics the beta cell loss in type 1 diabetes, from the significantly smaller loss of beta cells and insulin observed in the female mice, which mimic the beta cell loss anticipated in most cases of type 2 diabetes [31]. The data provide a significant step towards the validation of a probe monitoring the effect of candidate treatments in a pre-clinical setting, assuming that the expression of GLP-1rs is not altered under different glycaemic conditions [43]. Such pre-clinical studies should further benefit from the exquisite resolution achieved with high magnetic field MRI, which now allows for the visualisation of individual islets within an intact pancreas [21, 23].

Given that the data were generated using widely available MRI equipment, they also open exciting perspectives for the most-needed clinical imaging of patients with type 1 and type 2 diabetes. However, whether the method could improve the present status of human islet imaging [17, 22, 44] remains to be determined. MRI is a non-ionising technique that provides unsurpassed anatomical images of soft tissues, which facilitates the localisation of the signal source, but still features a rather low sensitivity and is hardly amenable to the quantification of a signal in absolute terms. These features are almost in direct contrast to those of nuclear imaging methods that provide for a modest anatomical resolution, require the use of radioactive probes and often require a co-registration by irradiating techniques such as computed tomography. Still, PET and SPECT have an exquisite sensitivity and are easily amenable to a precise quantitative evaluation of the signal [2, 3]. Accordingly, two recent studies using SPECT [17] and PET [45] have shown that the pancreatic signal correlates with beta cell mass in rodent models of diabetes. The authors of these studies also documented a large individual variation in the human pancreas signal [17, 44], which is consistent with the reported variable range of human beta cell mass [46]. Such a large interindividual variation calls for the repeated imaging of any given animal or patient, to obtain reliable evaluations over time. The radioactive dose used in nuclear imaging may potentially be a limiting factor in such longitudinal studies [8, 11, 14, 18, 19]. MRI may help to bypass this issue, as we now show that conditions can be defined to allow differentiation of maximal from submaximal alterations in beta cells content, and to sequentially and repeatedly image the same animal. Still, the MRI approach requires the use of contrast-enhancing agents ([21, 22, 30, 47], this study) to enhance the endocrine signal. As for the isotopes used in nuclear medicine imaging, the acute and long-term safety of these contrast-enhancing agents should be carefully evaluated [48, 49]. Furthermore, it remains to be determined whether MRI (as well as SPECT and PET) could detect, in the same individual or animal, smaller beta cell variations that those we reported here (about 50% in female RIP-DTr mice), which may be particularly important for the evaluation of new candidate treatments aimed at increasing the beta cell mass. Thus, no approach is presently fully specific for beta cell imaging. Multimodal imaging should be favoured to complement and coordinate the positive features of different methods, and a repeated longitudinal analysis of any given patient will most likely be required to obtain useful estimates of beta cell mass and function.

## Electronic supplementary material

Below is the link to the electronic supplementary material.

ESM Methods (PDF 80 kb)

ESM Fig. 1 (PDF 172 kb)

ESM Fig. 2 (PDF 59 kb)

ESM Fig. 3 (PDF 411 kb)

ESM Fig. 4 (PDF 1.05 mb)

ESM Fig. 5 (PDF 91.2 kb)

ESM Fig. 6 (PDF 89.5 kb)

## Abbreviations

DT: Diphtheria toxin
ExCys1: Modified exendin-4 with a cystein at position 1
ExScra: Random permutation of the exendin-4 peptide modified with a cystein at position 1
GLP-1r: Glucagon-like peptide 1 receptor
MTT: Thiazolyl blue tetrazolium bromide
Np647: Iron-oxide nanoparticle tagged with the fluorophore Alexa647
PET: Positron emission computed tomography
RIP-DTr: Rat insulin promoter-DT receptor
Sulfo-SMCC: Sulfosuccinimidyl-4-(*N*-maleimidomethyl) cyclohexane-1-carboxylate
SPECT: Single photon emission computed tomography
TE: Echo time
TR: Repetition time
TSE: Turbo spin echo
USPIO: Ultrasmall superparamagnetic particles of iron oxide
