# Supplementary material for: Targeting GLP-1 receptors for repeated magnetic resonance imaging differentiates graded losses of pancreatic beta cells in mice
Source: Diabetologia. 2014 Nov 22;58(2):304–12. doi: 10.1007/s00125-014-3442-2 (PMC4287680; doi:10.1007/s00125-014-3442-2)
Supplement: Supplementary file 1 — (PDF 80 kb) [file 125_2014_3442_MOESM1_ESM.pdf]

SUPPLEMENTARY METHODS:

In vitro studies.

Glucose-induced insulin secretion protocol: Cells were exposed for 24 h to 50 µg/ml Np647-ExCys1 added directly to the Dulbecco's modified Eagle's culture medium. Then, cells were washed with PBS, pre-incubated 30 min at 37°C in a KRB buffer, and eventually incubated for 30 min in the same medium supplemented with either 1.4, 8.4 or 16.8 mmol/l glucose. The medium was then collected, centrifuged for 20 min at 4°C and at 4000 rpm, and the supernatant frozen at -20°C for insulin release measurements. The cultures were kept overnight at 4°C in acid-ethanol, and the extracts were also frozen for determination of total insulin content. Insulin was measured by radioimmunoassay with a charcoal separation step, using rat insulin as standard and guinea pig anti-rat insulin serum as antibody (Linco Research, Inc.). Values of secreted insulin were expressed as percentage of the cell content.

KRB composition: 133.4 mmol/l NaCl, 5 mmol/l $NaHCO_3$, 4.7 mmol/l KCl, 1.2 mmol/l KH_2PO_4, 2.4 mmol/l $MgSO_4$, 3.4 mmol/l $CaCl_2$, 10 mmol/l Hepes, 2.8 mmol/l glucose and 0.5% BSA pH 7.4.

Dulbecco's modified Eagle's culture medium composition : 25 mmol/l glucose, and supplemented with 15% heat inactivated FCS, 70 µmol/l *β*-mercaptoethanol, 110 U/ml penicillin and 110 µg/ml streptomycin.
