# Supplementary material for: Targeting GLP-1 receptors for repeated magnetic resonance imaging differentiates graded losses of pancreatic beta cells in mice
Source: Diabetologia. 2014 Nov 22;58(2):304–12. doi: 10.1007/s00125-014-3442-2 (PMC4287680; doi:10.1007/s00125-014-3442-2)
Supplement: Supplementary file 2 — (PDF 172 kb) [file 125_2014_3442_MOESM2_ESM.pdf]

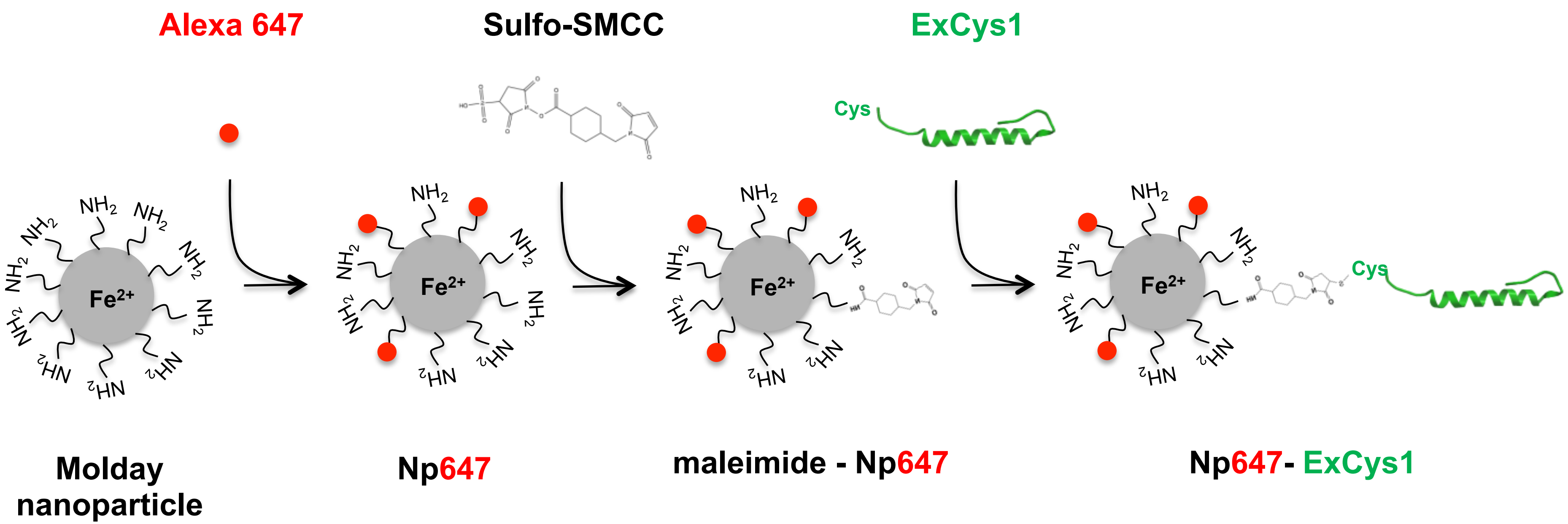

ESM Fig.1. Schematic view of the protocol for the production of the exendin-nanoparticle probe.
Iron oxide nanoparticles were chosen to decrease the MRI signal after specific targeting to beta cells by exendin-4, which binds to GLP-1R of the beta-cell membrane. To generate the probe, nanoparticles were first tagged with Alexa 647 for fluorescence localization, modified by the attachment of a sulfo-SMCC linker for binding the targeting peptide, and eventually reacted with an exendin-4 peptide, which was modified by addition of a Cys at position one, for targeting beta-cells. The same protocol was followed to synthesize the non-targeted nanoparticles, excepting that exendin-4 was replaced by the scrambled peptide (ExScra) of exendin-4.
