# Supplementary material for: Targeting GLP-1 receptors for repeated magnetic resonance imaging differentiates graded losses of pancreatic beta cells in mice
Source: Diabetologia. 2014 Nov 22;58(2):304–12. doi: 10.1007/s00125-014-3442-2 (PMC4287680; doi:10.1007/s00125-014-3442-2)
Supplement: Supplementary file 3 — (PDF 59 kb) [file 125_2014_3442_MOESM3_ESM.pdf]

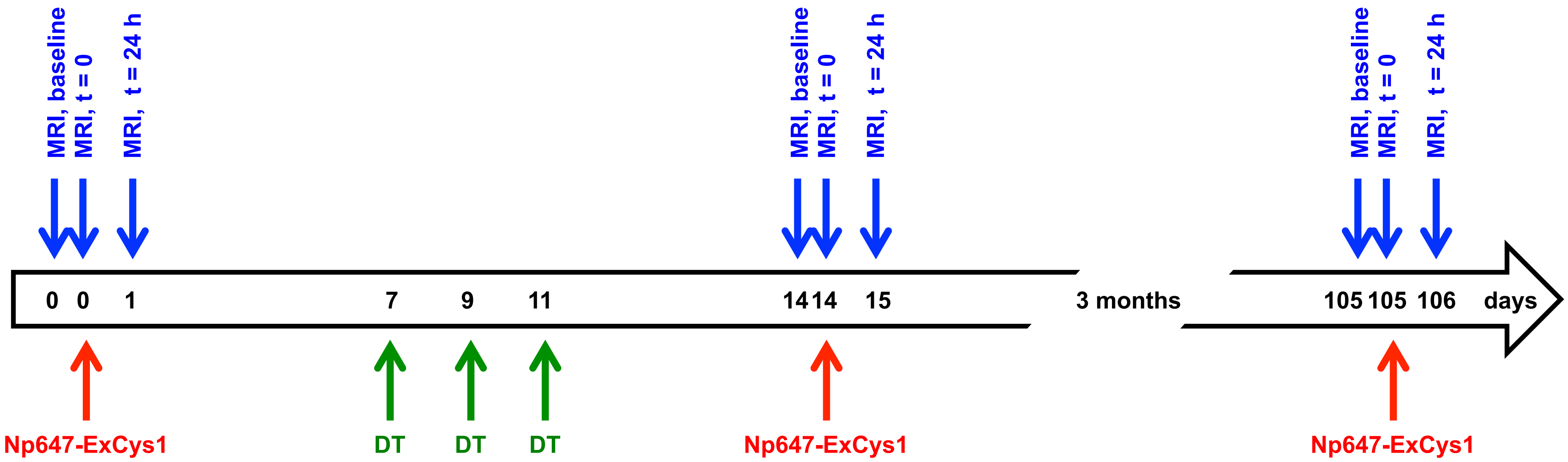

ESM Fig 2. Design of the *in vivo* experiments.
RIP-DTr mice were initially imaged by MR (blue arrows) prior to the injection of the probe, to obtain a baseline T2 value. They were then injected (still within the coil) through the retro-orbital venous plexus with 5 mg/kg Np647-ExCys1 (red arrows), and imaged again (time 0) for evaluation of the vascular volume fraction, which reflects the blood perfusion of pancreas. A third MRI scan was performed 24 h later, to allow for the evaluation of the probe accumulation. One week after this first imaging session, the very same mice were given 3 injections of diphtheria toxin (DT, green arrows) at 2 day intervals, to induce β-cell deletion. One week after the beginning of the DT administration, the mice underwent a second imaging session, as per the protocol of the first session. Female mice underwent a similar, third MRI session 3 months after the DT injection.
