# Supplementary material for: Targeting GLP-1 receptors for repeated magnetic resonance imaging differentiates graded losses of pancreatic beta cells in mice
Source: Diabetologia. 2014 Nov 22;58(2):304–12. doi: 10.1007/s00125-014-3442-2 (PMC4287680; doi:10.1007/s00125-014-3442-2)
Supplement: Supplementary file 4 — (PDF 411 kb) [file 125_2014_3442_MOESM4_ESM.pdf]

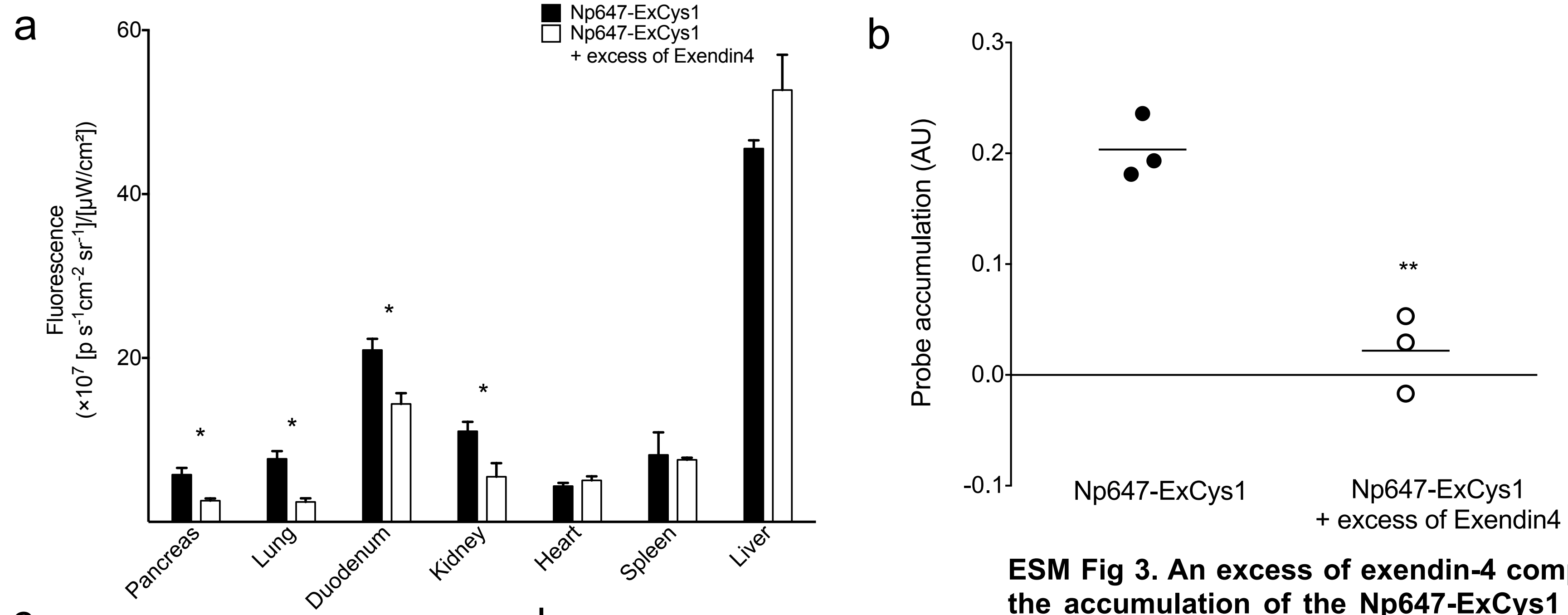

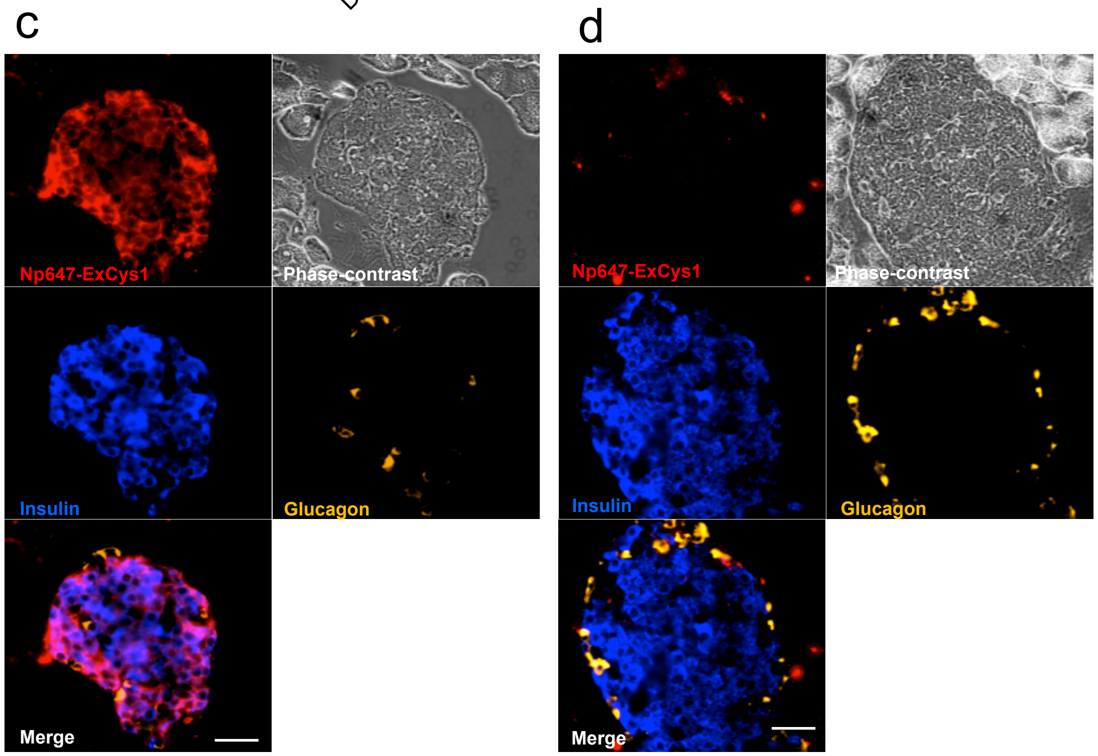

ESM Fig 3. An excess of exendin-4 competes with the accumulation of the Np647-ExCys1 probe. (a) Semi-quantitative analysis of fluorescence revealed a higher retention of Np647-ExCys1 in the absence (black bars) than in the presence of a 100 fold molar excess of exendin-4 (white bars) in organs known to express GLP-1rs, but not in those (spleen, liver) uptaking non-specifically the nanoparticles. Data are mean + SEM (n=3 per condition). * $p<0.05$, Np647-ExCys1 vs Np647-ExCys1 + excess of exendin-4. (b) The pancreas probe accumulation measured with MRI was also markedly decreased in the presence of an excess exendin-4. Symbols represent individual mice. Mean values are shown by the lines. ** $p<0.01$ vs Np647-ExCys1. (c) After the i.v. injection of Np647-ExCys1, fluorescence microscopy reveals that the exendin-targeted nanoparticles labeled the insulin-containing beta cells of RIP-DTr mouse. (d) Such a labeling was not observed in mice injected with Np647-ExCys1 in the presence of an excess of exendin-4. Bar, 30 µm.
