# Supplementary material for: Targeting GLP-1 receptors for repeated magnetic resonance imaging differentiates graded losses of pancreatic beta cells in mice
Source: Diabetologia. 2014 Nov 22;58(2):304–12. doi: 10.1007/s00125-014-3442-2 (PMC4287680; doi:10.1007/s00125-014-3442-2)
Supplement: Supplementary file 5 — (PDF 1.05 mb) [file 125_2014_3442_MOESM5_ESM.pdf]

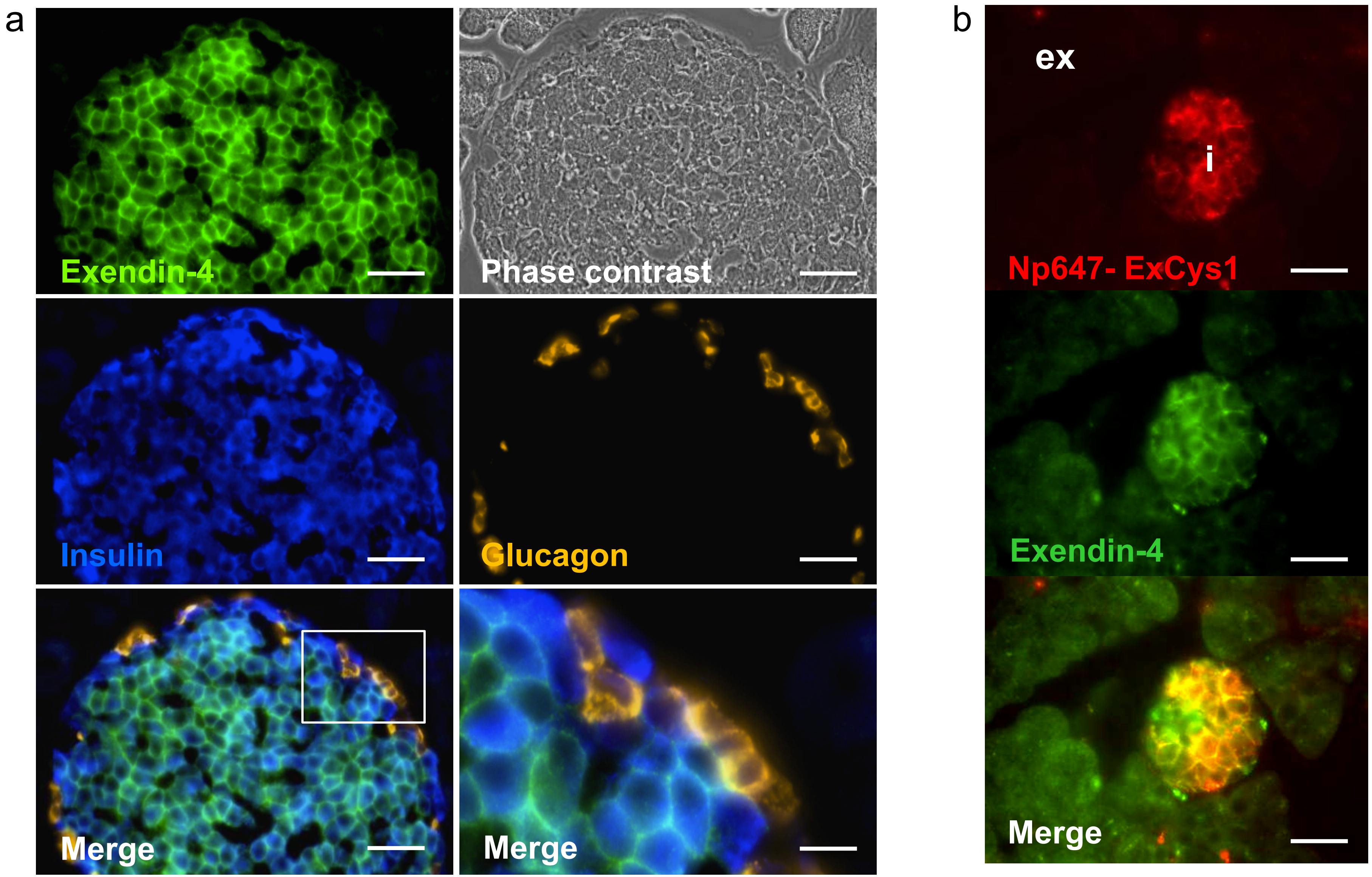

ESM Fig. 4. The Np647-ExCys1 probe colocalizes with exendin-4 *in vivo*. (a) One hour after the I.V. injection of 4 nmoles of exendin-4 peptide tagged with FITC on the K12 (Genicbio Limited, Shanghai, China), immunostaining shows that the fluoresceinated exendin-4 peptide distribution is limited to the insulin-containing beta cells, and excludes the glucagon-containing alpha cells. (b) After an i.v. injection of the Np647-ExCys1 probe, fluorescence microscopy reveals that the fluorochrome-tagged nanoparticles (upper panel) colocalizes with the attached exendin-4 peptide (as revealed by a antibody against exendin-4, middle and lower panel) on most islet cells (i). Bar, 30 μm in a, 50 μm in b, and 15 μm in the enlargement of the boxed area.
