# Supplementary material for: Targeting GLP-1 receptors for repeated magnetic resonance imaging differentiates graded losses of pancreatic beta cells in mice
Source: Diabetologia. 2014 Nov 22;58(2):304–12. doi: 10.1007/s00125-014-3442-2 (PMC4287680; doi:10.1007/s00125-014-3442-2)
Supplement: Supplementary file 6 — (PDF 91.2 kb) [file 125_2014_3442_MOESM6_ESM.pdf]

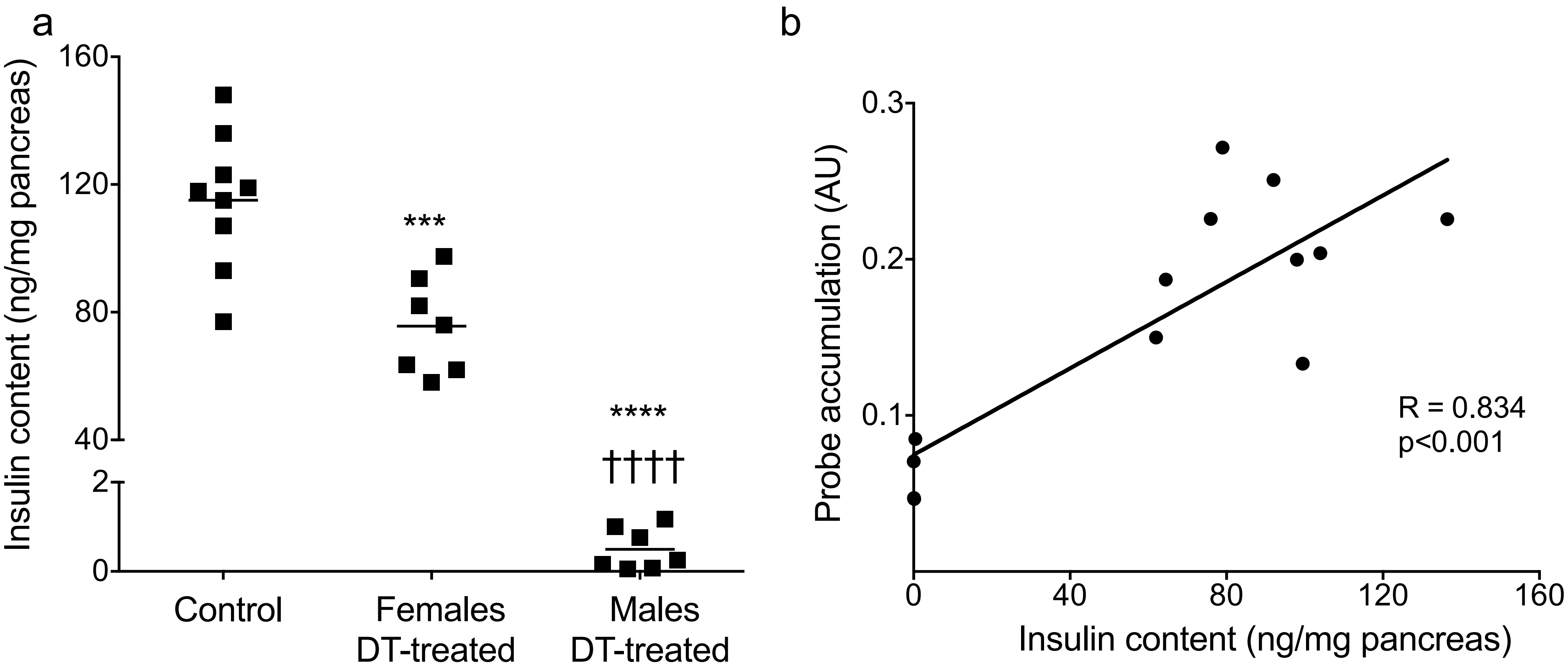

ESM Fig.5. The insulin content of pancreas is differentially altered by DT in male and female RIP-DTr mice, and correlates with the T2 MRI signal. (a) the insulin content of pancreas of male and female RIP-DTr mice was significantly reduced one week after the administration of DT, as compared to the control levels evaluated in animals which had not received DT. However, this reduction was much more severe in male than in female mice. Symbols represent individual mice, including several which had been imaged by MRI. Mean values are shown by the lines. ***$p<0.001$, ****$p<0.0001$ vs control values; $^{††††}p<0.0001$ vs DT-treated females. (b) the insulin content of pancreas correlated with the accumulation of the Np647-ExCys1 probe, as evaluated on T2-weighted images, in these animals whose pancreas could be sampled immediately after a MRI session.
