# Supplementary material for: Targeting GLP-1 receptors for repeated magnetic resonance imaging differentiates graded losses of pancreatic beta cells in mice
Source: Diabetologia. 2014 Nov 22;58(2):304–12. doi: 10.1007/s00125-014-3442-2 (PMC4287680; doi:10.1007/s00125-014-3442-2)
Supplement: Supplementary file 7 — (PDF 89.5 kb) [file 125_2014_3442_MOESM7_ESM.pdf]

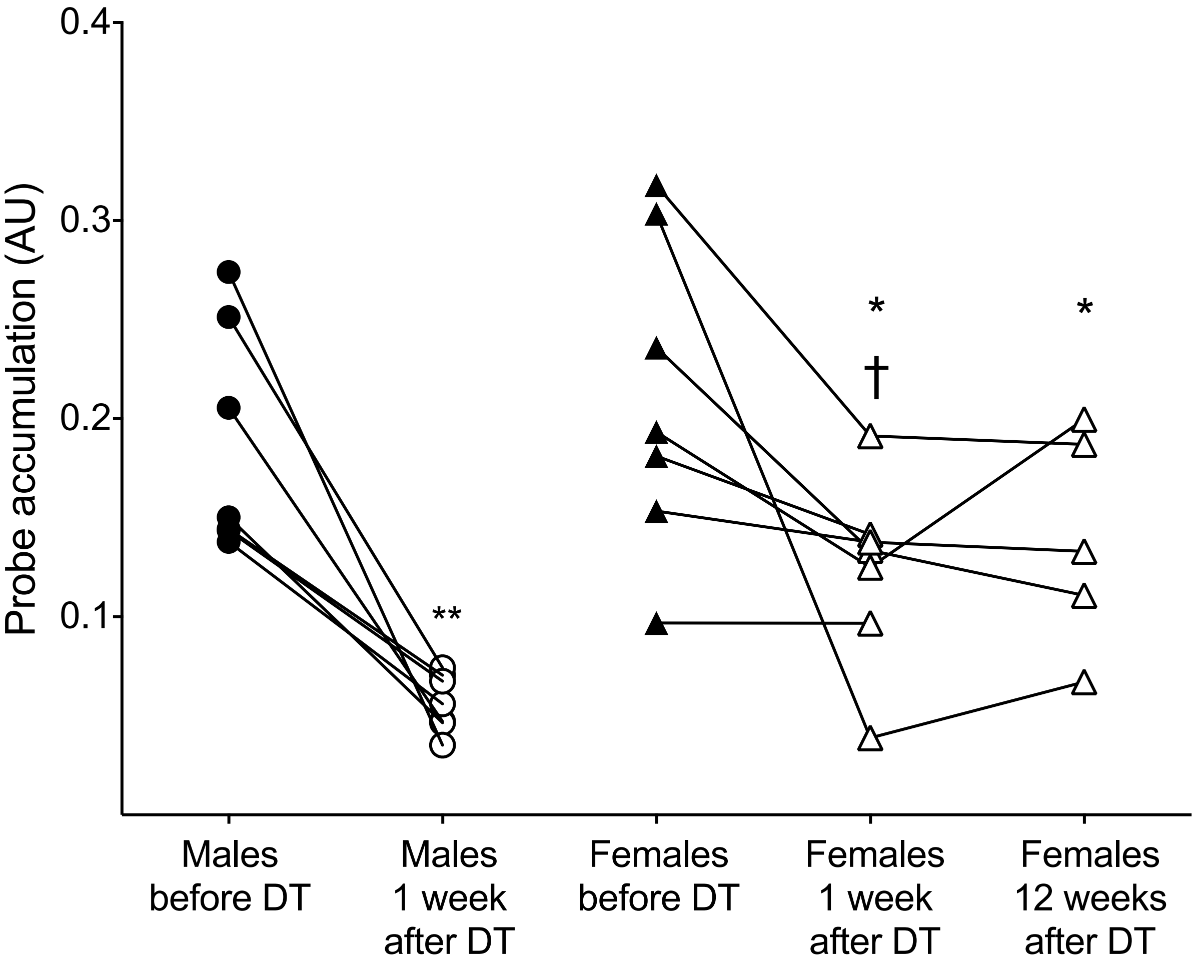

ESM Fig.6. The Np647-ExCys1 probe differentiates graded losses of beta cells in male and female RIP-DTr mice. Twenty four hours after the injection of Np647-ExCys1, similar amounts of the probe accumulated in the pancreas of control male (black circles) and female RIP-DTr mice (black triangles). One week after administration of DT, the accumulation of the probe dropped in the pancreas of most mice, of both genders. The drop was however significantly smaller in hemizygous females (white triangles) than in males (white circles). Symbols represent individual mice. The connecting black lines show the corresponding repeated measurements of individual mice. *$p<0.05$; ** $p < 0.01$ vs control of the same gender;. † $p<0.05$ vs DT-treated males.
